# Women and the Heart: Gender-Related Differences in Cardiovascular Care

**DOI:** 10.14797/mdcvj.1366

**Published:** 2024-03-14

**Authors:** Valeria E. Duarte

**Affiliations:** 1 Houston Methodist DeBakey Heart & Vascular Center, Houston Methodist, Houston, Texas, US

**Keywords:** pregnancy, congenital heart disease, arrhythmia in pregnancy, thoracic aortic disease, sex-specific etiologies, cardiomyopathy, pulmonary hypertension, estrogen paradox, ischemic heart disease

Despite advances in understanding and treatment, cardiovascular disease (CVD) continues to be the leading cause of death in women within the United States. And despite the now-common knowledge that as many women succumb to CVD as men, discrepancies still exist between the care that women receive.

Gender differences in women and men continue to permeate every aspect of cardiovascular health, from presentation and perception to inclusion in clinical trials, treatment, and outcomes. In 2017, this journal devoted an entire issue to women and heart disease. Seven years later, given the increased prevalence of CVD during pregnancy and its adverse effects even through the postpartum period, we present another issue on women and heart disease but with a focus primarily on cardio-obstetrics and with input from experts in this space.

We begin this issue with a review on hypertensive disorders of pregnancy, a topic that represents a first encounter with CVD for many women. Drs. Christopher Hobday, Courtney Newman, and coauthors provide an overview of diagnosing hypertensive diseases in pregnancy—a diagnosis that occurs in up to 16% of pregnant women. They describe key terms and types of hypertension and highlight the American College of Obstetricians and Gynecologists recommended guidelines for treatment.

Another pregnancy-related challenge—and a common cause of peripartum cardiovascular morbidity and mortality—is valvular heart disease. Typical hemodynamic shifts that occur in pregnancy can exacerbate existing cardiovascular issues and pose significant management challenges for clinicians. In “Valvular Heart Disease in Pregnancy,” coauthors Laith Alshawabkeh and Hilary Shapiro describe the normal hemodynamic changes of pregnancy and their effects on the most common valvular abnormalities, mechanical and bioprosthetic valve complications during pregnancy, and current management strategies.

With improved therapies for congenital heart disease (CHD) in children, more and more women with CHD are reaching childbearing age and becoming pregnant. Pregnant women with high-risk CHD present a complex clinical challenge. In “High-Risk Congenital Heart Disease in Pregnancy,” Drs. Carla Rodriguez and Saurabh Rajpal describe risk stratification and management of pregnant women with high-risk CHD, emphasizing the need to consider both anatomical and physiological complexity. They discuss how maternal physiological changes—such as blood volume increase, cardiac output changes, and alterations in vascular resistance—can significantly impact high-risk CHD patients, and they explain how management of high-risk CHD in pregnancy necessitates a multidisciplinary approach and individualized care.

In “An Overview of Arrhythmias in Pregnancy,” Drs. Kamala Tamirisa, Estefania Oliveros, and colleagues look at another complication of pregnancy: cardiac arrhythmias. Prevalent and causing concern for both maternal and fetal health, arrhythmias during pregnancy are increasing due to advances in congenital heart surgery and a growing number of pregnant women with structural heart disease. An increasing prevalence of more serious arrhythmias requires a comprehensive, multidisciplinary approach involving specialists throughout various stages of pregnancy.

Our next article looks at the risks and challenges of thoracic aortic disease (TAD) during pregnancy, particularly for women with genetic syndromes like Marfan syndrome, Loeys-Dietz syndrome, and vascular Ehlers-Danlos syndrome. In “The Impact of Pregnancy in Patients with Thoracic Aortic Disease: Epidemiology, Risk Assessment, and Management Considerations,” authors Valeria Duarte, Jessica Magny, and Michael Singh describe a multidisciplinary approach to assessment and medical management and explain its importance when navigating the complexities of TAD in pregnancy and for improving maternal and neonatal outcomes.

Next we look at “Cardiomyopathies in Women” by Dr. Cindy Martin. While the annual rate of heart failure mortality is lower in females than in males, more women die from heart failure each year due to its increased overall incidence in females. Several anatomical differences in the female heart affect both systolic and diastolic cardiac physiology, yet women remain notably underrepresented in clinical trials. Sex-specific etiologies of heart failure and unique manifestations in genetic-related cardiomyopathies also lead to significant sex-related differences for women’s access to and outcomes in advanced heart failure therapies.

Pulmonary hypertension (PAH) is a rare and devastating disease that is seen in registries across the globe. Despite the higher incidence in women, women with PAH are found to have better outcomes than men, a finding that has been labeled the “estrogen paradox.” In their review on PAH in women, authors Zeenat Safdar and Eunwoo Park describe the effects specific to women with PAH and stress that this population should be given special consideration for sexual health, contraception, family planning, and treatment before, during, and after pregnancy. While pregnancy outcomes have improved over the years, pregnant women with PAH should be referred to a pulmonary hypertension care center and follow a multidisciplinary team approach.

We then shift to ischemic heart disease (IHD), the leading cause of morbidity and mortality in both genders. Outcomes for IHD show that young women fare the worst, a likely reflection of the more complex range of IHD attributed to female sex hormones. In their review titled the “Spectrum of Ischemic Heart Disease Throughout the Women’s Life Cycle,” Drs. Karla Kurrelmeyer, Smitha Narayana Gowda, and Sai sita Garapati touch on the various presentations of IHD throughout a woman’s life, share insights on the diagnosis and management of IHD, and highlight where further randomized controlled studies are needed to determine optimal treatment and inform guideline-directed medical therapy.

Our next review, “Primary Prevention of Cardiovascular Disease in Women,” provides a bird’s eye view of the reasons CVD remains a leading cause of mortality in women. Authors Khurram Nasir, Eleonora Avenatti, and colleagues discuss recent guidelines for primary prevention of CVD and how the increasing prevalence of female-specific risk enhancers substantially affect future risk occurring throughout a woman’s lifetime. They also stress the need for innovative strategies to improve primary prevention of CVD in women.

Wrapping up this issue, Drs. Gladys Velarde, Madeline Mahowald, and coauthors offer more insights and suggest some solutions in “Sex Disparities in Cardiovascular Disease.” Their article starts with key facts on how and why CVD in women remains underdiagnosed, undertreated, and portends worse outcomes than in men. After summarizing differences in cardiovascular risk factors and disparities in management and outcomes of IHD, heart failure, aortic stenosis, and atrial fibrillation, the authors propose strategies to overcome these disparities and offer examples of successful programs that other providers can model.

Through the articles presented in this issue, our aim is to underscore the profound impact of CVD on women across their lifespan, with a particular emphasis on the heightened risks during pregnancy and strategies to tailor management to the challenges each of the described conditions bring. The critical necessity for establishing multidisciplinary teams to deliver specialized clinical care and to propel research forward is consistently emphasized throughout this issue.

We earnestly hope that ongoing research initiatives, coupled with preventive measures and counseling efforts in clinical practice, alongside educational campaigns, will pave the way for improved outcomes and a more equitable standard of care for women.

We are most grateful to our experts for sharing their invaluable insights, all in the pursuit of enhancing awareness regarding the distinct manifestations and ramifications of CVD in women. We hope that our readers will find these reviews informative, thought-provoking, and helpful in your clinical practice.

## Guest Editor Biography

The editors of the *Methodist DeBakey Cardiovascular Journal* express our appreciation to Dr. Valeria Duarte for her guidance, insight, and dedication in curating this issue on woman and the heart.

## Valeria E. Duarte, MD, MPH

**Figure:**
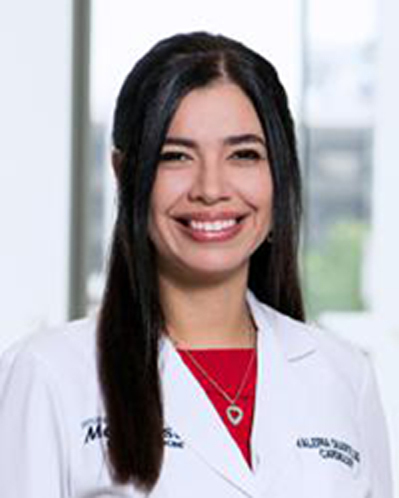


Dr. Valeria Duarte received her medical degree at Favaloro University in Buenos Aires and completed her Master’s in Public Health at the Harvard T.H. Chan School of Public Health. Her training includes internal medicine residency at the Cleveland Clinic Foundation followed by cardiology training at the University of Florida. Dr. Duarte further specialized in adult congenital heart disease (ACHD) and pulmonary hypertension through a fellowship at Boston Children’s, Brigham and Women’s, and Massachusetts General Hospital, where she was awarded a 2-year T-32 National Institute of Health grant. She subsequently remained on the faculty at Boston Children’s and Brigham and Women’s and as an instructor at Harvard Medical School. Dr. Duarte joined the Houston Methodist DeBakey Heart & Vascular Center in September 2019, where she leads the ACHD imaging, ACHD transplantation, and cardio-obstetrics programs. Her clinical and research interest areas are advanced cardiac imaging in ACHD, high-risk pregnancy in patients with cardiovascular disease, and genetic aortopathies.

